# Evaluation of 99Tcm-DTPA orbit SPECT/CT combined with thyroid function test in the treatment of radioactive iodine I-131 in patients with thyroid-associated ophthalmopathy-hyperthyroidism

**DOI:** 10.5937/jomb0-48734

**Published:** 2024-11-16

**Authors:** Li Su, Ping Mi, Wenqiang Niu, Ting Zhou, Wang Yang, Cheng Chen, Chenggang Huang

**Affiliations:** 1 The Central Hospital of Xiaogan, Department of Nuclear Medicine, Xiaogan City, Hubei Province, China

**Keywords:** 99Tcm-DTPA, orbital SPECT/CT, radioactive iodine I-131, thyroid-associated ophthalmopathy, clinical activity score, 99Tcm-DTPA, orbitalni SPECT/CT, radioaktivni jod I-131, oftalmopatija povezana sa štitastom žlezdom, klinički aktivni skor

## Abstract

**Background:**

Thyroid-associated ophthalmopathy (TAO) is an autoimmune response to inflammation of the thyroid and orbital tissue. This research evaluated the efficacy of 99Tcm-DTPA orbital SPECT/CT combined with thyroid function test in radioactive iodine I-131 (RAI) treatment of TAO-hyperthyroidism.

**Methods:**

We retrospectively studied clinical activity score (CAS), blood thyrotropine (TSH), free triiodothyronine (FT3), free thyroxine (FT4), thickness of extra-ocular muscle (EOM), and uptake rate (UR) of 99Tcm-DTPA orbital SPECT/CT of 43 patients after 6 months of treatment with 20 mCi RAI. Parameters were compared before and after RAI in patients assessed as effectively treated (normal thyroid function or hypothyroidism), and correlations between blood FSH, FT3, FT4, thickness of EOM, and UR were analyzed after treatment.

**Results:**

After RAI, 35 cases (70 eyes, 81.4%) had normal or hypothyroidism, and 8 cases (16 eyes, 18.6%) had hyperthyroidism. Compared with the patients who failed treatment, effectively treated patients had lower CAS, FT3, FT4, and UR and higher blood TSH. In patients with effective treatment, UR of the inferior rectus muscle was positively correlated with FT3 and FT4. Adverse RAI outcomes were associated with smoking and higher iodine-thyroid iodine uptake before treatment.

**Conclusions:**

Combined with TSH, FT3, and FT4 levels, the reduction of 99Tcm-DTPA orbital SPECT/CT UR also indicates an improvement in the disease course of patients. The UR of the inferior rectus muscle can be an objective index to evaluate the curative effect of TAO patients.

## Abstract (sr)

**Uvod:**

Oftalmopatija povezana sa štitastom žlezdom (TAO) je autoimuni odgovor na upalu tkiva štitaste žlezde i orbitalnog tkiva. Ovo istraživanje se bavilo efikasnošću 99Tcm-DTPA orbitalnog SPECT/CT kombinovanog sa testom funkcije štitaste žlezde u lečenju TAO-hipertireoze radioaktivnim jodom I-131 (RAI).

**Metode:**

Retrospektivno smo proučavali klinički aktivni skor (CAS), nivo tirotropina (TSH) u krvi, slobodni trijodtironin (FT3), slobodni tiroksin (FT4), debljinu ekstraokularnih mišića (EOM) i stopu preuzimanja (UR) 99Tcm-DTPA orbitalnog SPECT/CT kod 43 pacijenta nakon 6 meseci lečenja sa 20 mCi RAI. Parametri su upoređeni pre i posle RAI kod pacijenata koji su procenjeni kao uspešno lečeni (normalna funkcija štitaste žlezde ili hipotireoza), a korelacije između nivoa TSH, FT3, FT4 u krvi, debljine EOM i UR su analizirane nakon lečenja.

## Introduction

Thyroid-associated ophthalmopathy (TAO) is the most common extra-thyroid organ autoimmune disease and the most common extra-thyroid manifestation of Graves’ disease. Its signs and symptoms include eyelid retraction, exophthalmos, strabismus, vision loss, extra-ocular muscle (EOM) hypertrophy, and soft tissue involvement, as well as typical imaging changes [1] [2]. TAO is the most common adult orbital disease at present, and the pathogenesis of TAO has not yet been fully elucidated [3]. TAO can occur with hyperthyroidism, hypothyroidism, and normal thyroid function [4]. The course of TAO is mainly divided into the active inflammatory stage and quiescent fibrotic stage [5]. The principle of treatment is to treat the primary disease and maintain normal thyroid function actively. For active patients, drug therapy (including hormones and immunosuppressants) or radioactive iodine-131 (RAI) therapy is mostly referred to, while for quiescent patients, surgical treatment is mostly considered [6].

Single photon emission computed tomography/ computed tomography (SPECT/CT) is a radionuclide imaging test. Combining the advantages of SPECT and CT, it can accurately image and suggest lesion-related information [7]. 99 Tcm-diethylenetri-aminepentaacetic acid (DTPA) is a sensitive marker of inflammatory activity. The accumulation of 99mTc-DTPA in the orbital soft tissues is proportional to the activity of inflammation, which may explain the high uptake of 99mTc-DTPA in TAO due to inflammation [8]. 99Tcm-DTPA orbital SPECT/CT tomography fusion imaging can evaluate the active and quiescent stages of TAO, providing semi-quantitative information on disease activity [9] [10]. Clinical studies have confirmed that immunosuppressive agents and local orbital radiotherapy affect active TAO [11].

Thyroid function tests mainly include total tri-iodothyronine (T3), total thyroxine (T4), free T3 (FT3), free T4 (FT4), and thyrotropine (TSH) [12]. FT3 and FT4 exclude the binding of some T3 and T4 hormones with thyroid-binding globulin, which can more accurately display the actual situation of the thyroid. TSH level is considered to be a first-line indicator reflecting the function of the hypothalamic-pituitary-thyroid axis [13]. Therefore, the detection of these three indicators can determine hyperthyroidism or hypothyroidism more accurately [14].

This study selected patients who were assessed as moderate-to-severe active TAO according to the European Group of Graves’ Orbitopathy (EUGOGO) classification and clinical activity score (CAS) and retrospectively reviewed their pre-treatment SPECT/CT, thyroid function test indicators, and post-treatment evolution to evaluate the efficacy of SPECT/CT combined with thyroid function test in RAI treatment of TAO-hyperthyroidism.

## Materials and methods

### Research objects

The study protocol followed the Declaration of Helsinki, and the Ethics Committee of The Central Hospital of Xiaogan approved the study population and clinical evaluation. All patients and controls were enrolled after providing and signing written informed consent to the imaging procedure and to participate in the anonymous analysis. Data were retrospectively collected from 78 patients who received 99mTc-DTPA SPECT/CT between January 2021 and September 2023. The patients were diagnosed with TAO according to the 2021 EUGOGO guidelines [15].

Inclusion criteria: (1) Male or female patients aged 18–80 years; (2) patients with moderate to severe active TAO [16]. Ocular features were graded and evaluated according to the EUGOGO guidelines and CAS, meeting one or more of the following criteria: eyelid retraction 2 mm, moderate to severe soft tissue invasion resulting in EOM involvement; exophthalmos 20 mm, intermittent or persistent diplopia (the Gorman diplopia scale was used to score the diplopia) [17]; CAS 3 in one or both eyes; (3) patients who intend to undergo and receive RAI therapy; (4) patients with 24-hour radioiodine uptake (RAIU) >40%; patients who stopped antithyroid drugs or other medications that may interfere with thyroid hormone metabolism at least 1 month before 24-hour RAIU test.

Exclusion criteria: (1) pregnant or lactating patients; (2) patients receiving GC or any immuno-suppressive drug within the previous 3 months; (4) patients with suspected malignant thyroid nodules; (5) patients with a history of thyroidectomy; (6) patients with optic neuropathy. Finally, 43 subjects, 25 women and 11 men (86 eyes in total), aged 23–64 years, were included.

### Thyroid mass assessment

Thyroid mass was assessed in all patients prior to RAI treatment. Thyroid size measurements were performed independently by 2 radiologists using the GE Logiq 400 Pro ultrasound system (GE Healthcare, Wauwatosa, WI) and a 10 MHz probe. Longitudinal and transverse scans were performed to measure the depth (D), width (W), and length (L). The average of three measurements of thyroid D, W, and L was used to determine thyroid volume (V): 0.479 × 3D × W × l (cm). The thyroid volume is the sum of the volumes of the two thyroid lobes, and isthmus volume is ignored. Thyroid mass is calculated by multiplying the volume by 1.0 g/cm^3^ (density of thyroid tissue).

### RAI uptake

All patients underwent 2-hour and 24-hour RAIU examinations prior to RAI treatment. Patients received a 5 mCi dose of sodium iodide (131I-NaI) on an empty stomach. RAI uptake was measured 24 hours after administration using the FH-458 Thyroid Uptake Test system (CNNC Nuclear Instrument Co., Ltd., Beijing, China). For the uptake measurement, the NaI crystal scintillation probe (3 cm in diameter) was placed at the thyroid at a fixed distance of 25 cm for 2 min and at the thigh at the same distance for 2 min to obtain background correction. The activity per minute count on the thyroid was compared with the activity measured from a standard containing 185KBq (5mCi) 131I.

### Orbital tomography fusion imaging acquisition

99mTc-DTPA 740MBq (20mCi) was injected into the patient’s elbow vein. Orbital CT and SPECT were performed 20 min later. The patient was supine on the examination bed with the head fixed and eyes closed. Scanning was performed with a dual-head SPECT/CT scanner (Priority 16, Philips, Netherlands) using low-energy and high-resolution collimators. The field of view of the probe was from the top of the head to the lower edge of the occipital bone. CT scan was performed first (The slice thickness of the CT scan was 0.625 mm, the pitch ratio was 0.938:1, the voltage was 120 kV, and the current was 250 mA). SPECT tomography was performed at the same location, equipped with a low-energy, high-resolution collimator with a magnificence of 1 and a collection matrix of 128 × 128. The energy window opened ±10 % at 140 keV, 20 s/frame, 60 frames per probe. Finally, fusion images were obtained based on CT and SPECT images. Images of patients before and 6 months after RAI treatment were collected.

### SPECT/CT imaging measurements

SPECT/CT results were reviewed by 2 experienced nuclear medicine specialists. (1) Measurement of EOM thickness: The horizontal diameters of the medial rectus (MR) and lateral rectus (LR) and the vertical diameters of the superior rectus (SR) and inferior rectus (IR) were measured on a series of images. The average diameter of MR, LR, SR, and IR was 4.0 mm, 3.4 mm, 3.6 mm, and 4.2 mm, respectively. (2) The thickness of EOM was defined as the region of interest (ROI), and semi-quantitative analysis was performed using ROI technology. DTPA uptake was determined by manually placing circular ROI on SPECT images after CT attenuation correction. The ROI on the occipital lobe of the same layer was selected to determine the background uptake value. (3) Uptake rate (UR) was calculated as the ratio of average EOM uptake to average occipital imaging agent uptake in the same layer. SPECT/CT imaging parameters were measured before and 6 months after RAI treatment. UR greater than 1.2 was considered positive.

### Laboratory measurement

Before RAI treatment, 3–5 mL cubitus venous blood was collected, centrifugation was performed at 3000 r/min for 20 min, and TSH, TRAbs, T3, T4, FT3, and FT4 were determined by electrochemiluminescence instruments (Roche, Basel, Switzerland).

### Follow-up after RAI treatment

Patients were examined 3 to 6 months after RAI. A complete medical history should be taken at every visit, and a physical exam should be performed. Three thyroid functions were detected during reexamination, including FT3, FT4, and TSH. Other tests were performed as needed. Effective remission of hyperthyroidism is defined as clinical and laboratory evidence of normal thyroid function or stable hypothyroidism in the absence of thyroid hormone medications after 6 months of RAI treatment. If the hyperthyroidism persists or recurs, the treatment is considered to have failed. Normal values: TSH (0.27–4.2 μIU/mL), FT3 (3.1–6.8 pmol/L), and FT4 (12–22 pmol/L).

### Sample size estimation

Utilize G*Power software version 3.1.9.2 to determine the necessary sample size for the study. Employ a significance level of 0.05 ( =0.05), a power of 80% ( =0.20), and an effect size of 0.8 for bilateral testing. Anticipate a failure rate of 20–30% for the initial treatment. The calculated sample size for the successful treatment group is estimated to be 60–64 cases (eyes), while the failure group is projected to consist of 16 –19 cases (eyes).

### Statistical analysis

Enumeration data were expressed as n (%), and statistical significance was assessed using Chi-square or Fisher’s exact test. The distribution of continuous variables was evaluated using the Kolmogorov-Smirnov test, with results expressed as mean ±SD or median (quartile). Student-t test or Mann-Whitney U test were used to compare independent samples between the two groups. Three or more independent samples were compared using one-way ANOVA or Kruskal-Wallis tests. Wilcoxon Signed Rank test was used to compare the samples of the two groups with non-normal distribution. Spearman method was used to calculate the correlation between variables, and P<0.05 was corrected by Benjamini-Hochberg’s false discovery rate with adjusted P<0.05 representing statistical significance. Statistical tests were performed using SPSS 22.0, and graphs were produced using GraphPad Prism 9.0.

## Results

### Laboratory and clinical characteristics of patients at baseline and before RAI treatment

A total of 43 patients were enrolled, including 28 men and 15 women (total 86 eyes), with a median age of 39.6 years. As shown in Table 1, the mean 2-hour RAIU for these patients was 50.84±8.99%, and the 24-hour RAIU was 78.17±16.1%. After 6 months of RAI treatment, 81.4% of patients were in remission, including 8 cases of hyperthyroidism (18.6%), 26 cases of normal or stable hypothyroidism (60.5%), and 9 cases of acquired hypothyroidism (20.9%). Among them, the preoperative thyroid mass of patients with effective treatment was significantly lower than that of patients with failed treatment (P=0.034). Patients after treatment were divided into thyroid hyperfunction, normal or stably decreased function, and decreased function. The patients’ pre-treatment laboratory measurements of RAI were then compared according to the grouping. After RAI treatment, there were no statistically significant differences in TSH, TRAbs, T3, T4, FT3, FT4, TG-Ab, TPO-Ab, and thyroid mass in patients (P>0.05). However, smokers and patients previously treated with ATD were still diagnosed with hyperthyroidism after RAI treatment in a higher proportion than the other two groups.

**Table 1:** Clinical characteristics and measurement results of patients before baseline and RAI treatment. RAI treatment, radioactive 131 iodine therapy; BMI, Body Mass Index; TAO, thyroid-associated ophthalmopathy; ATD, Antithyroid drugs; TSH, thyroid stimulating hormone; TRAbs, serum anti-TSH receptor autoantibodies; T3, Triiodothyronine; T4, Tetraiodothyronine; FT3, free triiodothyronine; FT4, free thyroxine; RAIU, Radioactive iodine uptake. Count variables were analyzed using the Chi-square or Fisher’s test. The comparison of three independent samples was conducted using one-way ANOVA or Kruskal-Wallis, and P<0.05 was considered a significant difference.

Variable	Hyperthyroidism<br>(n=8)	Euthyroidism/Stable<br>decline (n=26)	Hypothyroidism<br>(n=9)	*P* value
Age, years	39.9 (30.6–56.5)	37.5 (27.6–47.9)	39.6 (29.3–43.5)	0.455
Gender, M:F	3:5	19:7	6:3	0.181
BMI, kg/m^2^	19.74 (18.37–20.55)	20.5 (18.2–23.8)	20.1 (19.2–21.7)	0.854
Smoking habits (Yes)	5	3	2	0.011
History of TAO (Yes)	5	9	3	0.204
Previous ATD treatment (Yes)	5	5	1	0.036
Pre-TSH, μIU/mL	0.122±0.058	0.109±0.079	0.117±0.067	0.665
Pre-TRAbs, IU/mL	4.87 (4.46–8.28)	5.68 (2.92–9.60)	4.68 (3.72–8.68)	0.759
Pre-T3, ng/mL	7.38 (6.96–8.67)	7.54 (5.64–10.24)	8.48 (5.35–9.19)	0.912
Pre-T4, ng/mL	21.58 (17.87–27.69)	22.12 (18.72–27.27)	18.78 (17.41–24.39)	0.652
Pre-FT3, pmol/L	16.59 (11.95–17.69)	13.46 (9.21–20.33	14.24 (13.3–16.03	0.815
Pre-FT4, pmol/L	34.14 (23.58–39.53)	31.56 (25.65–35.31	32.49 (28.13–37.11)	0.648
2-hour RAIU, %	60.39 (53.54–66.13)	50.30 (44.79–53.56)	51.19 (44.88–53.5)	0.019
24-hour RAIU, %	77.93 (68.52–88.22)	78.85 (69.69–83.79)	81.25 (71.33–89.76)	0.988
2/24-hour RAIU	0.72 (0.71–0.84)	0.65 (0.56–0.73)	0.63 (0.58–0.72)	0.023
Thyroid mass (g)	39.3 (34.67–44.03)	33.69 (25.26–39.88)	35.33 (26.60–41.88)	0.034

### Treatment outcomes and effectiveness of RAI

**Figure 1:**
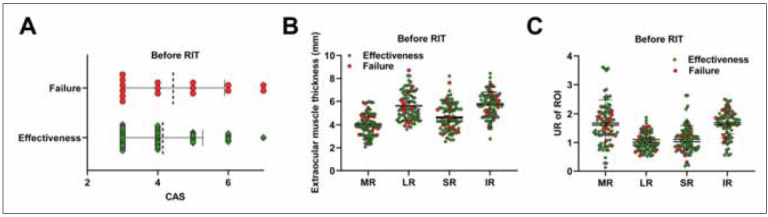
Patients were divided into effective and failed groups based on their post-treatment outcomes. Comparison of (A) baseline CAS, (B) EOM thickness, and (C) UR. CAS: clinical activity score; MR, medial rectus muscle; LR, lateral rectus muscles; SR, superior rectus muscle; IR, inferior rectus muscle; UR, ROI uptake rate. Mann-Whitney U test was used between the two groups. Three or more groups were tested using the Kruskal-Wallis test, P>0.05.

**Figure 2:**
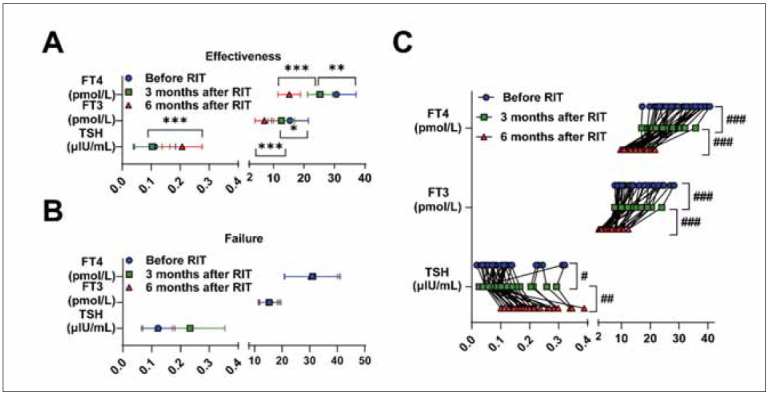
Changes in blood thyroid function in patients after RAI treatment. (A) Changes in TSH, FT3, and FT4 in the blood of patients in the effective and failed groups before and after RAI treatment. The significance test between two independent samples was conducted using Mann-Whitney U test, * P<0.05, ** P<0.01, *** P<0.001. (B) Changes in TSH, FT3, and FT4 in the blood of paired samples in the effective group. The significance test of two paired samples was conducted using Wilcoxon matched pairs signed rank test. # P<0.05, ## P<0.01, ### P<0.001. RIT, radioiodine treatment.

**Figure 3:**
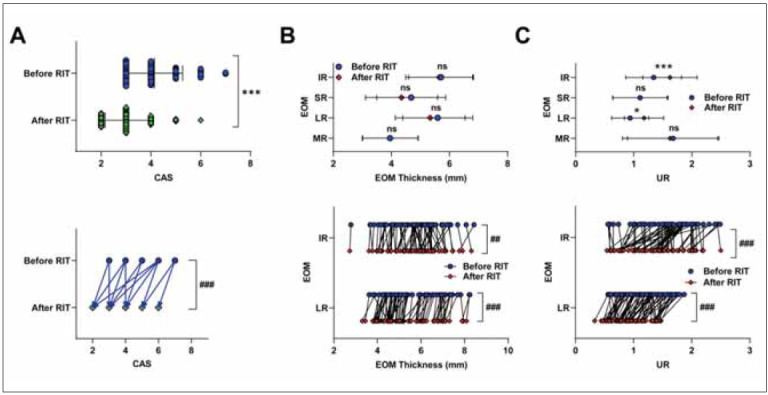
CAS and orbital SPECT/CT related parameters of effective patients after 6 months of RAI treatment. Comparison of (A) CAS, (B) EOM thickness, and (C) UR of effective patients before and after treatment. CAS: clinical activity score; MR, medial rectus muscle; LR, lateral rectus muscles; SR, superior rectus muscle; IR, inferior rectus muscle; UR, uptake rate of ROI. The significance test between two independent samples was conducted using Mann-Whitney U test, * P<0.05, ** P<0.01, *** P＜0.001. The significance test of two paired samples was conducted using Wilcoxon matched pairs signed rank test, ## P<0.01, ### P<0.001.

Of the 43 patients (86 eyes) treated with RAI, 35 (70 eyes) responded to treatment. After 6 months of RAI treatment, patients were divided into the effective group (70 eyes) and the failed group (16 eyes) according to treatment effectiveness. As shown in Table 2 and Figure 1, there were no significant differences in CAS, EOM thickness, and UR between the two groups at baseline based on post-treatment outcomes. As shown in Table 3, after 6 months of RAI treatment, TSH was increased, and FT3 and FT4 were decreased in the effective group compared to the failed group (P<0.001). Furthermore, we observed a stable recovery of these measures after treatment in the effective group but not in the failed group (Figure 2A). Notably, TSH, FT3, and FT4 also recovered gradually and steadily at 3 and 6 months after treatment (Figure 2B). As expected, the median CAS was lower in the effective group than in the failed group (P<0.001). After RAI treatment, UR in the LR and IR were significantly decreased in the effective group compared with the failed group (P < 0.05, P<0.001). However, we did not find statistical differences in EOM thickness and UR of the MR and SR between the two groups (P>0.05). As shown in Figure 3A-C, subgroup analysis was conducted among effective patients. Compared with before treatment, CAS and UR of the MR and SR were all decreased in patients in the effective group 6 months after treatment (P<0.001), and no decrease in EOM thickness was observed in the failed group (P>0.05). For patients in the effective group, we also found reductions in CAS and thickness and UR of IR and SR (P<0.001).

**Table 2:** CAS, EOM thickness, and UR of patients before RAI treatment. CAS, Clinical Activity Score; CAS: Clinical Activity Score; MR, internal rectus muscle; LR, external rectus muscle; SR, superior rectus muscle; IR, inferior rectus muscle; UR, uptake rate of ROI. Mann-Whitney U test was used between the two groups, and P<0.05 was considered a significant difference.

Variable	Effectiveness (70 eyes)	Failure (18 eyes)	*P* value
CAS	4 (3–5)	4 (3–5.25)	0.579
Thickness, mm			
MR	3.92 (3.19–4.50)	4.14 (3.38–4.72)	0.723
LR	5.27 (4.61–6.59)	5.54 (4.98–5.96)	0.997
SR	4.36 (3.73–5.56)	4.39 (3.68–5.32)	0.718
IR	5.73 (4.86–6.46)	5.79 (5.16–6.50)	0.752
UR			
MR	1.62 (1.25–2.18)	1.67 (1.39–1.86)	0.892
LR	0.94 (0.77–1.15)	1.16 (0.84–1.37)	0.536
SR	1.06 (0.82–1.35)	1.05 (0.76–1.25)	0.923
IR	1.69 (1.38–1.90)	1.75 (1.48–1.99)	0.891

**Table 3:** Comparison of thyroid function, CAS, and orbital SPECT/CT imaging-related parameters between effective and failed patients receiving RAI treatment after 6 months. CAS, Clinical Activity Score; CAS: Clinical Activity Score; MR, internal rectus muscle; LR, external rectus muscle; SR, superior rectus muscle; IR, inferior rectus muscle; UR, uptake rate of ROI. Mann-Whitney U test was used between the two groups, and P<0.05 was considered a significant difference.

Variable	Effectiveness (n=3)	Failure (n=8)	P value
Thyroid Blood measurement			
TSH, μIU/mL	0.18 (0.16–0.25)	0.11 (0.08–0.13)	0.001
FT3, pmol/L	7.61 (4.49–8.95)	15.68 (13.52–16.58)	0.001
FT4, pmol/L	15.68 (11.87–17.68)	30.69 (23.79–38.56)	
Thyroid mass (g)	25.65 (20.69–30.52)	39.13 (33.02–42.78)	0.001
	(70 eyes)	(18 eyes)	
CAS	3 (2–3)	4 (3–5.25)	0.001
Thickness, mm			
MR	3.94 (3.16–4.49)	4.10 (3.48–4.76)	0.853
LR	5.01 (4.48–6.22)	5.54 (4.98–5.96)	0.368
SR	4.1 (3.36–5.33)	4.44 (3.65–5.42)	0.731
IR	5.85 (4.82–6.47)	5.69 (5.09–6.5)	0.953
UR			
MR	1.56 (1.05–2.16)	1.67 (1.35–1.85)	0.777
LR	0.90 (0.62–1.19)	1.21 (0.86–1.37)	0.048
SR	1.06 (0.81–1.35)	1.01 (0.84–1.24)	0.785
IR	1.35 (0.98–1.67)	1.64 (1.27–2.04)	0.011

**Figure 4:**
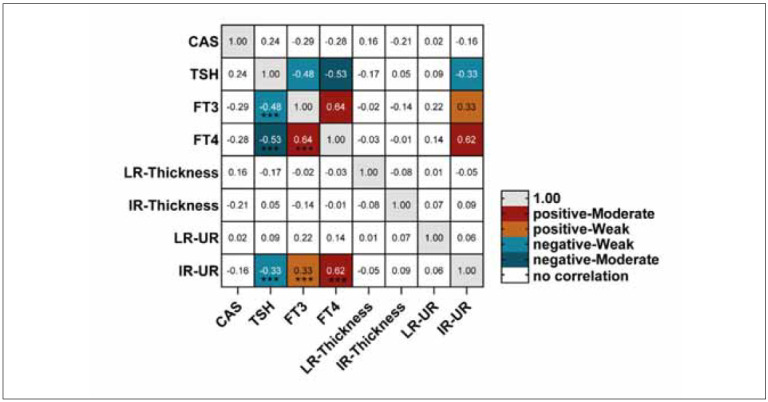
The correlation between CAS and thyroid function indicators, LR, IR thickness, and UR in effective patients treated with RAI for 6 months. The Spearman method was used to calculate the correlation between variables, and the Benjamin Hochberg false discovery rate analysis was performed to calculate the corrected P value for P<0.05. Adjusted P<0.05 had statistical significance.

Next, based on the effectiveness of the above treatment, Spearman correlation analysis showed that TSH was significantly negatively correlated with FT3, FT4, and UR of IR at a weak to moderate intensity (P<0.001). In addition, we also observed a weak and moderate positive correlation between UR of IR and FT3 and FT4, respectively (P<0.001), and no significant correlation between EOM thickness and corresponding UR was found (P>0.05). Finally, smoking (OR 1.13; 95%CI 1.04–1.58; P=0.026) and 2/24- hour RAIU (OR 1.05; 95%CI 1.01–1.36; P=0.001) were independent predictors of RAI treatment effectiveness (Table 4). Figure 4

**Table 4:** Univariate and multivariate logistic regression analysis for predicting treatment response. OR, odds ratio; CI, Confidence Interval. P<0.05 was considered a significant difference.

Variables	Univariate			Multivariate		
	OR	95% CI	P value	OR	95% CI	P value
Age (years)	0.73	0.62–1.25	0.265			
Gender (%)	1.05	0.56–3.15	0.891			
Smoking habits (Yes)	1.73	1.03–2.35	0.001	1.13	1.04–1.58	0.026
ATD treatment (Yes)	0.86	0.32–3.25	0.827			
2/24-hour RAIU, %	1.59	1.09–2.48	0.001	1.05	1.01–1.36	0.001
Thyroid mass (g)	1.06	0.79–3.68	0.658			

## Discussion

At present, there is still a lack of objective and unified criteria for the evaluation of the therapeutic effect of TAO, and the clinical efficacy of the same treatment measures in different studies is very different due to the influence of factors such as case selection, treatment method, and evaluation criteria. Therefore, it is necessary to establish an effective method to evaluate the clinical efficacy of TAO. RAI has been used in the treatment of autoimmune hyperthyroidism for more than 60 years [18]. The best treatment for thyroid dysfunction in China and its effect on eye diseases remain to be confirmed. A randomized clinical trial conducted in patients with moderate-to-severe active Graves’ disease showed better clinical efficacy after receiving RAI alone [19]. Our study showed that the EOM UR and thyroid function tests (TSH, FT3, and FT4) based on 99Tcm-DTPA orbital SPECT/CT imaging could better evaluate the treatment effect of patients with RAI. In addition, 2/24-hour RAIU predicted RAI outcomes in patients, suggesting that early iodine-thyroid uptake is important for RAI outcomes.

CAS is the most commonly used clinical scale to assess the activity of TAO. However, it has some limitations in reflecting ocular surface inflammation and is somewhat subjective. Nuclear medicine imaging has broad application prospects in this field. In the past two decades, studies have mainly focused on nuclide-labelled somatostatin receptor imaging, which relevant guidelines have recommended because of its ideal results [20]. In addition, orbital CT can provide information on exophthalmos and muscle enlargement, which is also meaningful for diagnosis. In our current study, patients with CAS 3 were selected, and the median diameter of LR (5.34 mm), SR (4.36 mm), and IR (5.75 mm) in EOM was greater than the average diameter of the Chinese population. Similar to the results in a previous study, the orbital muscles in TAO were enlarged, especially the MR and IR [21]. In addition, we found that the median UR in the MR (1.66) and IR (1.69) was greater than 1.2 (positive), suggesting that UR has the potential to assess inflammatory infiltration within EOM.

The EUGOGO recommends that all patients with thyroid dysfunction recover and maintain normal thyroid function in a timely manner [15]. Therefore, in our study, we mainly evaluated blood thyroid function indicators (FSH, FT3, and FT4 levels) of patients after RAI treatment. After 6 months of RAI treatment, thyroid function stabilized at 81.4% (35 patients in 70 eyes) and mostly returned to normal. It has been reported that thyroid mass is a predictor of success with the RAI fixed-dose approach [22] [23]. Our study showed that patients who were rated as responding to treatment had lower thyroid mass than those who failed, both before and after treatment. However, thyroid mass was not observed as a predictor of RAI treatment success through multifactorial logic analysis. As expected, CSA was reduced in patients who responded to RAI treatment. CAS is judged based on the ocular signs and symptoms of the anterior segment, while SPECT/CT reflects the activity of the posterior orbital segment, the main site of inflammatory deposition [24]. Orbital inflammation is a basis for reflecting the course of TAO disease [25]. To further evaluate the orbital inflammation in TAO patients after treatment, we evaluated the EOM thickness and SPECT/CT imaging results of the patients and found that there was no difference in EOM thickness between the effective group and the failed group and only observed a decrease in the UR of IR in the effective group in positive SPECT/CT imaging (>1.2). However, the thickness of the IR and SR of the effective group was reduced. This indicates that EOM thickness and UR have a certain correlation in individual treatment. To this end, we used Spearman correlation analysis to understand better the association of these indicators with RAI treatment effectiveness. Our data showed that the UR of IR had a weak and moderate positive correlation with FT3 and FT4, respectively. This suggests that orbital UR, FT3, and FT4 are important for predicting the outcome of RAI treatment.

The EUGOGO also mentioned that the prevalence of TAO in smokers is higher than that in nonsmokers, and smokers are more likely to experience treatment failure or progression progression after receiving RAI therapy [15]. Studies have shown that the higher the thyroid iodine uptake, the faster the turnover of iodide in thyroid cells, which shortens the residence time of therapeutic 131I in the gland, leading to treatment failure [26] [27] [28]. Consistent with these reports, we found that smokers and high iodine-thyroid uptake (2/24-hour RAIU) were predictors of treatment affecting RAI treatment outcomes. RAI treatment were followed for at least another 6 months to rule out transient hypothyroidism. Second, this was a retrospective study, and data on postoperative thyroid autoantibody titers, especially TRAb levels, were lacking. TRAb may help predict the severity of the disease and the likelihood of treatment failure. In addition, samples from TAO patients in The Central Hospital of Xiaogan who were treated with RAI were not representative of the whole.

## Conclusions

For patients with TAO-hyperthyroidism treated by RAI, the reduction of 99Tcm-DTPA orbital SPECT/CT imaging UR also indicated that the disease course of the patients was improved based on TSH, FT3, and FT4 levels. The UR of SR can be used as an objective index to evaluate the curative effect of TAO patients.

## Dodatak

### Acknowledgements

Not applicable.

### Funding

Not applicable.

### Availability of data and materials

The datasets used and/or analyzed during the present study are available from the corresponding author upon reasonable request.

### Ethics approval

The present study was approved by the Ethics Committee of The Central Hospital of Xiaogan, and written informed consent was obtained from all patients prior to the start of the study. All procedures were performed in accordance with the ethical standards of the Institutional Review Board and The Declaration of Helsinki, as well as its later amendments or comparable ethical standards.

### Authors’ contributions

Li Su designed the research study. Ping Mi and Wenqiang Niu performed the research. Chenggang Huang and Ting Zhou provided help and advice on the experiments. Wang Yang, Cheng Chen and Chenggang Huang analyzed the data. Li Su wrote the manuscript. Li Su and Chenggang Huang reviewed and edited the manuscript. All authors contributed to editorial changes in the manuscript. All authors read and approved the final manuscript.

### Conflict of interest statement

All the authors declare that they have no conflict of interest in this work.
